# Functionalized Peptide Fibrils as a Scaffold for Active Substances in Wound Healing

**DOI:** 10.3390/ijms22083818

**Published:** 2021-04-07

**Authors:** Justyna Sawicka, Emilia Iłowska, Milena Deptuła, Paweł Sosnowski, Piotr Sass, Katarzyna Czerwiec, Klaudia Chmielewska, Aneta Szymańska, Zuzanna Pietralik-Molińska, Maciej Kozak, Paweł Sachadyn, Michał Pikuła, Sylwia Rodziewicz-Motowidło

**Affiliations:** 1 Department of Biomedical Chemistry, Faculty of Chemistry, University of Gdańsk, 80-308 Gdańsk, Poland; justyna.sawicka@ug.edu.pl (J.S.); aneta.szymanska@ug.edu.pl (A.S.); 2 Department of Organic Chemistry, Faculty of Chemistry, University of Gdańsk, 80-308 Gdańsk, Poland; emilia.ilowska@ug.edu.pl; 3 Laboratory of Tissue Engineering and Regenerative Medicine, Department of Embryology, Medical University of Gdańsk, 80-210 Gdańsk, Poland; milenadeptula@gumed.edu.pl (M.D.); klaudia.chm@gmail.com (K.C.); 4 Laboratory for Regenerative Biotechnology, Faculty of Chemistry, Gdańsk University of Technology, 80-233 Gdańsk, Poland; paw.sosno@gmail.com (P.S.); piotrsass@gmail.com (P.S.); psach@pg.edu.pl (P.S.); 5 Department of Clinical Anatomy, Medical University of Gdańsk, 80-210 Gdańsk, Poland; katarzyna.czerwiec@gumed.edu.pl; 6 Department of Macromolecular Physics, Faculty of Physics, Adam Mickiewicz University in Poznań, 61-712 Poznań, Poland; zuzannap@amu.edu.pl (Z.P.-M.); mkozak@amu.edu.pl (M.K.)

**Keywords:** peptide fibrils, scaffolds, CD, AFM, TEM, cell proliferation, fibroblasts, keratinocytes, wound healing in vivo, regeneration

## Abstract

Technological developments in the field of biologically active peptide applications in medicine have increased the need for new methods for peptide delivery. The disadvantage of peptides as drugs is their low biological stability. Recently, great attention has been paid to self-assembling peptides that can form fibrils. Such a formulation makes bioactive peptides more resistant to enzymatic degradation and druggable. Peptide fibrils can be carriers for peptides with interesting biological activities. These features open up prospects for using the peptide fibrils as long-acting drugs and are a valid alternative to conventional peptidic therapies. In our study, we designed new peptide scaffolds that are a hybrid of three interconnected amino acid sequences and are: pro-regenerative, cleavable by neutrophilic elastase, and fibril-forming. We intended to obtain peptides that are stable in the wound environment and that, when applied, would release a biologically active sequence. Our studies showed that the designed hybrid peptides show a high tendency toward regular fibril formation and are able to release the pro-regenerative sequence. Cytotoxicity studies showed that all the designed peptides were safe, did not cause cytotoxic effects and revealed a pro-regenerative potential in human fibroblast and keratinocyte cell lines. In vivo experiments in a dorsal skin injury model in mice indicated that two tested peptides moderately promote tissue repair in their free form. Our research proves that peptide fibrils can be a druggable form and a scaffold for active peptides.

## 1. Introduction

**Figure 1:**
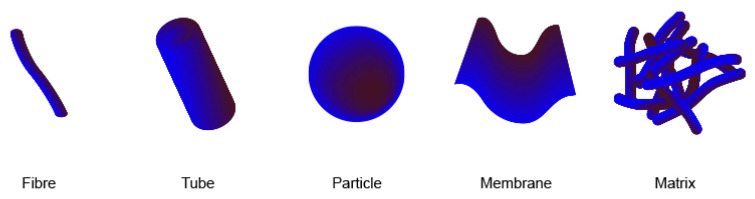
Types of nanomaterials used in the drug delivery system based on peptides. Based on [4].

The widely observed tendency, in nature, of different types of molecules to self-assemble has inspired a particular scientific interest in studying this process. One of the areas of this research is addressed to the utilization of self-assembling peptides as functional nanomaterials [1,2,3]. These peptide nanomaterials can adopt different shapes, divided into the five following groups: fibrillar, tubular and particulate with more complex variations, including membranes and matrices (Figure 1).

Depending on what shape self-aggregating peptides take, they may have different applications. For example, *N*-protected dipeptides like Fmoc-FF-COOH or Boc-FF-COOH [5,6,7] have been used to produce functional peptide nanotubes (Figure 1—tube) for casting molds of metal nanowires or electrochemical biosensing platforms [3,8,9]. Most often, due to the content of bioactive and biocompatible amino acids in their composition, self-assembling peptides are used in medical applications. For example, the FF dipeptide nanotube was tested as a drug delivery material. Silva et al. applied this approach by loading the FF nanotubes with fluorescent rhodamine B as a model drug to study the kinetics of its release as well as cytotoxicity [10]. It was also shown that these peptide nanotubes possess low cytotoxicity due to their high biocompatibility. Another example is the self-assembly scaffold (RADA)_4_. This peptide, composed of 16 amino acid residues, has a high tendency toward self-association in an aqueous solution at high concentration (~10 mg/mL) and forms stable, twisted β-sheet fibrils that further assemble in the form of a hydrogel (Figure 1—matrix) [11,12]. This hydrogel’s properties, such as stable structures and nanofibers in water, offer a new perspective toward using it as a scaffold for biologically active substances [13,14]. It has been reported that human fibroblasts cultured in (RADA)_4_ hydrogels show chondrogenic potential with significant upregulation of proteoglycan aggrecan expression [15]. In addition, (RADA)_4_ hydrogels have been used to promote differentiation of stem cells by providing an extracellular matrix (ECM)-like support [16,17].

Peptide fibers constitute one of the most numerous and important naturally occurring assemblies [18,19,20]. Multiple peptides with different amino acid sequences spontaneously form these highly stable and well-organized assemblies under various conditions. The potential applications of peptide fibrils often outweigh those of synthetic polymers, as they can fulfill a biological function in addition to their mechanical properties [21,22]. Their high endurance, structure, strength and resistance to many physical and chemical factors, together with their high biocompatibility and biodegradability, open up possibilities to use such fibrils as pharmaceuticals, in bioengineering and in nanotechnology [23,24,25]. In addition to remarkable stability, peptide fibrils can be reservoir sources of bioactive peptides, and their structure in many cases may provide a controlled and slow release of a peptide with confirmed biological activity. These features open up prospects for using amyloid fibrils as functional nanostructures in a bottom-up fabrication method for biomedical applications [26]. Peptide fibrils, as well as peptide hydrogels and peptide nanotube drug delivery systems, potentially have wide applications in medicine. In the literature, there are several examples in which scaffolds with different applications were made of peptides arranged in fibrils [17,27,28]. An example is gonadotropin-releasing hormone (*p*-Glu-His-Trp-Ser-Tyr-Gly-Leu-Arg-Pro-Gly-NH_2_; GnRH) and its analogs, which have been studied as potential drugs against, e.g., prostate cancer or breast cancer [29]. Currently, Degarelix^®^ is in commercial use as a peptide fibril drug formed of a gonadotropin-releasing hormone antagonist for the treatment of prostate cancer [30].

As shown above, self-assembling peptides as compounds with unique structural and biological properties can be considered a promising solution for peptides to be druggable and more applicable in modern medicine [1,2,3]. In this paper, we describe new peptide fibrils in the form of a scaffold consisting of fibrillogenic and pro-regenerative fragments. The fibrillogenic part of the designed peptides forms peptide fibrils and can be used as a nanomaterial or as a carrier (scaffold) for a biologically active peptide sequence. Peptides designed in this way, i.e., forming fibrils, constitute a natural reservoir of slowly released active sequence, cleaved from the fibrils by enzymes present in their environment. Such peptides can also be slowly released from the fibril as individual peptide molecules containing a biologically active part in its sequence. In order to check whether the peptides we designed have fibrillogenic properties and whether peptide fibrils can be a scaffold for an active sequence, we performed a series of experiments, including: structural studies (circular dichroism (CD), thioflavin test, transmission electron microscopy (TEM), atomic force microscopy (AFM); stability tests in water and human plasma; and tests in cellular (lactate dehydrogenase (LDH)assay, cell proliferation assay (XTT) and collagen synthesis) and animal models (injury of dorsal skin in mice).

## 2. Results

### 2.1. Structural Studies

#### 2.1.1. Peptide Fibril Design

**Figure 2:**
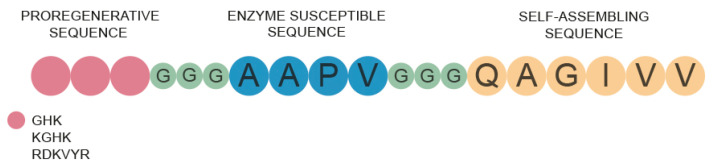
Scheme of the peptide scaffold construct.

The aim of our project was to develop new peptide scaffolds and to test their physicochemical and biological properties in the field of skin wound regeneration. For this purpose, we designed peptide constructs that were a hybrid of three interconnected sequences, namely, a self-assembling QAGIVV fragment, named FC in this work corresponding to the steric zipper sequence from a human protein—cystatin C, with shuffled residues to ensure high fibrillization propensities [31,32]; AAPV, which is sensitive to neutrophil elastase; and biologically active RDKVYR [33] or KGHK or GHK [34,35,36,37] sequences, which show propensities toward skin wound regeneration. The individual segments were connected by linkers consisting of three glycine residues (Figure 2). All designed sequences of the peptides are presented in Table 1. All compounds were thoroughly characterized (Figure S1) and for the next experiments only products with a purity exceeding 98% were used.

#### 2.1.2. Three-Dimensional Fibrillar Scaffold Formation

**Figure 3:**
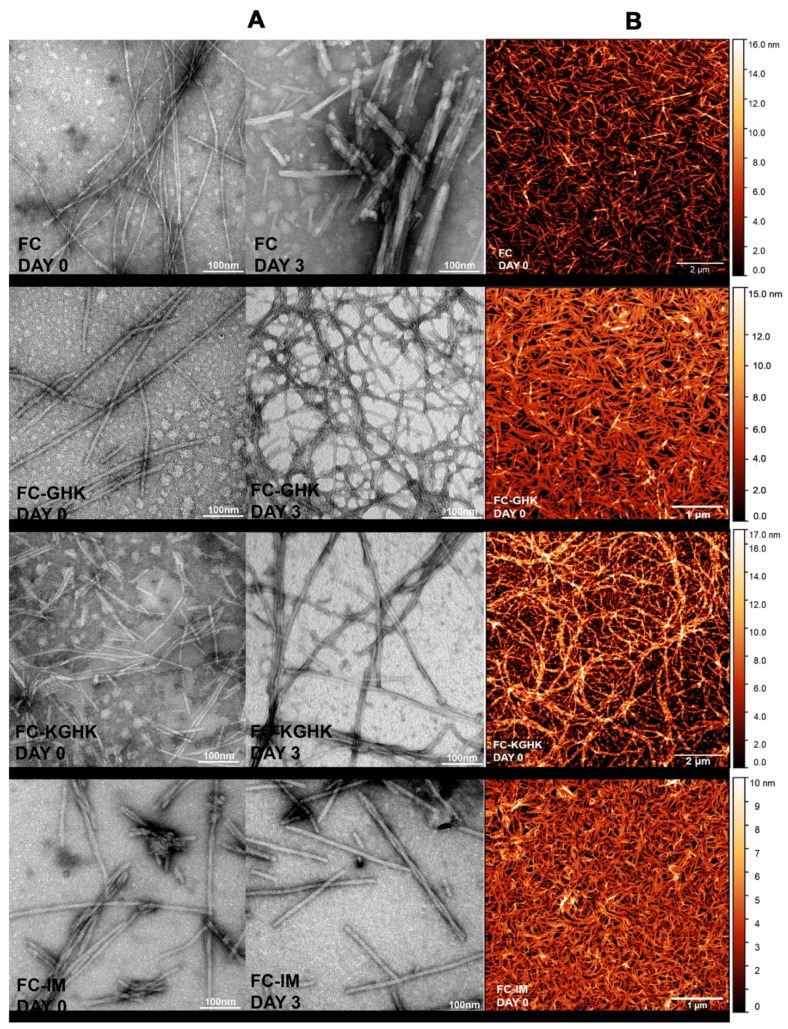
(**A**) Transmission electron micrographs (TEM) for peptides FC, FC-GHK, FC-KGHK and FC-IM taken before and on the 3^rd^ day of incubation in PBS buffer; (**B**) Atomic force microscopy (AFM) images of fibrils taken briefly after dissolution in PBS buffer.

Two complementary microscopic imaging techniques, namely, transmission electron microscopy (TEM) and atomic force microscopy (AFM), were used to visualize and compare the morphology of the obtained fibrils. As can be seen in TEM images all the studied compounds form fibrils directly after dissolution in PBS buffer (Figure 3A, left). There are some visible differences in fibril morphology, with FC-GHK fibrils being shorter but most closely resembling the fibrils formed by the unmodified fibrillogenic FC peptide, and the fibrils formed by the FC-KGHK and FC-IM peptides being slightly thicker (10–12 nm). The TEM micrographs clearly show that the peptide fibrils twist around each other and form characteristic larger fibers.

The high fibrillization propensity of the studied compounds was further confirmed by a picture obtained from AFM measurement of samples briefly after their dissolution (Figure 3B). In comparison to TEM, this imaging method offers more precise detection of the height of the obtained fibrils. The average heights of the studied fibrils were in a range up to 10–14 nm. The AFM pictures also show formation of a dense three-dimensional network of peptide fibrils that completely covers the mica surface, despite the small amount of sample applied and extensive washing of the mica surface. Such a dense, entangled network is especially visible for the FC-GHK and FC-IM peptides. The formed fibers had a length of 0.5 to 2 µm.

#### 2.1.3. Thioflavin T Assay

**Figure 4:**
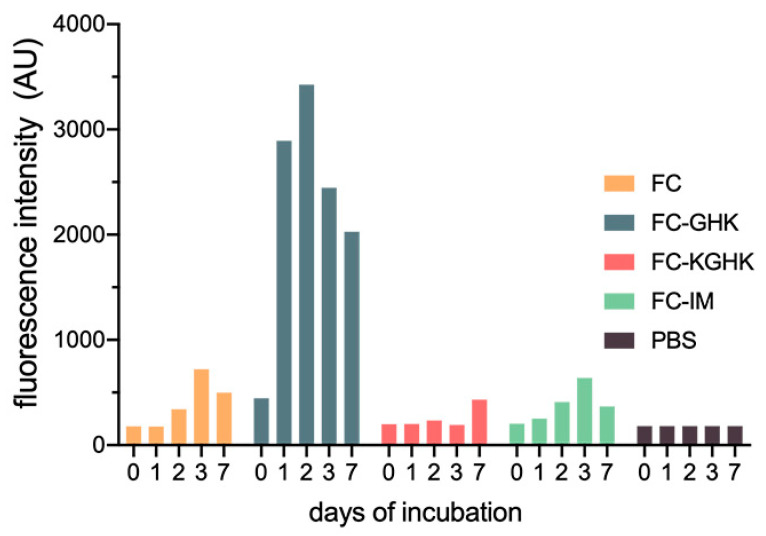
Dependence of the fluorescence intensity of the peptide-thioflavin T complex on the time of incubation, relative to a control (PBS solution).

Analysis was carried out to confirm the presence of β-structures that form a complex with thioflavin T, which gives a characteristic emission spectrum at 482 nm. The most interesting results were obtained for the FC-GHK compound, which showed a significant increase in the ThT emission intensity, suggesting effective sample fibrilization during the first two days of incubation (Figure 4). After this time, there was gradual precipitation of the peptide from the solution, which began to form a gel. For the FC peptide and two analogs, namely, FC-KGHK and FC-RDKVYR, an increase in ThT fluorescence intensity was much slower and required at least three days of incubation (Figure 4). The observed fluctuations of the reporter dye fluorescence intensity were, most likely caused by the precipitation (Figure S2) of peptide fibrils from the solution and the formation of new amounts of these fibrils. The presented results confirmed the ability of the designed peptides to form fibrils, albeit with different kinetics and efficiency.

#### 2.1.4. Circular Dichroism Analysis

**Figure 5:**
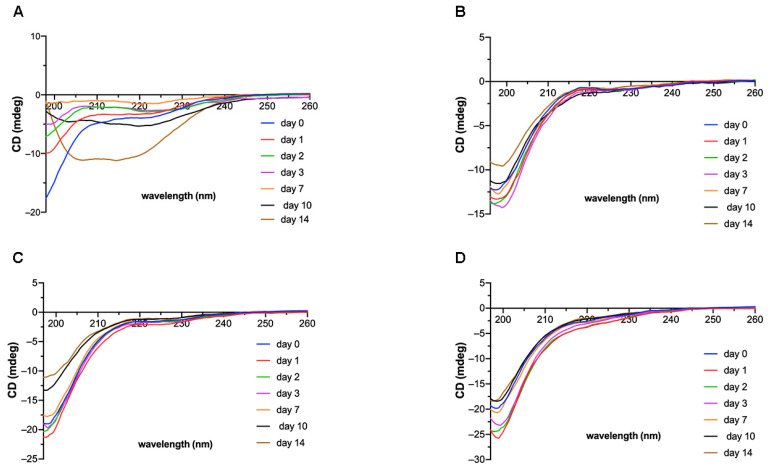
Circular dichroism (CD) spectra for the peptides (**A**) FC, (**B**) FC-GHK; (**C**) FC-KGHK and (**D**) FC-IM over 14 days incubation at a concentration of 1 mg/mL in PBS.

Circular dichroism (CD) analysis was used to monitor the time-dependent changes of the secondary structure content (Figure 5). These experiments were technically difficult because of precipitation of the peptides from the PBS solution during the incubation time, even at early stages (Figure 5A). Therefore, before the CD measurements, all samples were centrifuged and the CD spectra of a clear solution were then measured.

The CD spectra show that, at the beginning of incubation, the FC peptide adopted a random coil conformation. The small shallow minimum at 220 nm indicates the presence of a β-sheet conformation. Over the incubation time, the content of the β-sheet structure increased and finally, at the 14th day of incubation, a deep minimum at λ ≈ 215 nm, indicating the formation of a β-sheet structure, was observed (Figure 5A, gold line). The CD spectra of the FC-GHK (Figure 5B), FC-KGHK (Figure 5C) and FC-IM (Figure 5D) peptides indicate the existence of a disordered structure (deep min at λ ≈ 200 nm), both at the beginning and after 14 days of incubation. FC-GHK and FC-KGHK initially formed a random coil conformation but small conformational changes were observed (slight changes in the value of mdeg at λ ≈ 220 nm). TEM and AFM techniques clearly showed fibril formation by the FC-GHK, FC-KGHK and FC-IM peptides, but the CD spectra showed a random coil structure, in contrast to the FC peptide. The reason for this could be the presence of a non-fibrillogenic and highly flexible fragment containing six glycine residues. The proportion of the peptide’s disordered part, relative to the fibrillar part, which locally forms the β-structure, was very high. This was manifested in the CD spectrum by a spectral curve typical of a random coil structure.

### 2.2. Stability Tests

#### 2.2.1. Release of the Active Sequence

**Figure 6:**
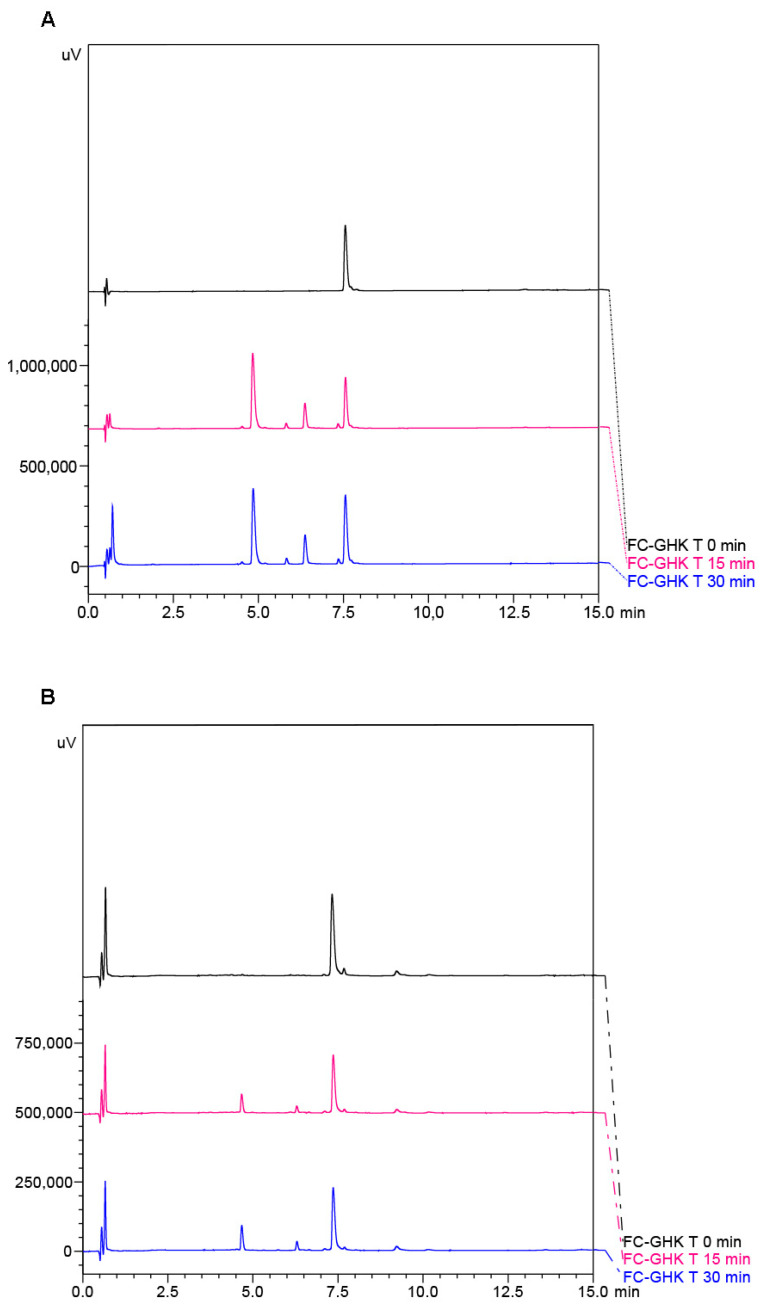
Chromatograms for (**A**) non-fibrillated FC-GHK and (**B**) FC-GHK as the fibril form, after neutrophil elastase digestion at the start (black line), after 15 min (pink line) and after 30 min (blue line) of incubation with the enzyme.

For the peptides with active sequences, the release of the active sequences was tested. The peptides were incubated with human neutrophilic elastase and were then analyzed by chromatography (RP-HPLC) (Figure 6A) and mass spectrometry (MS) (Figure S3). After 30 min of incubation, a decrease in the peak intensity of the substrates was observed, along with the appearance of new peaks from new digestion products. MS analysis showed that all the studied peptides were cleaved at the expected position between residues V and G, after the elastase-specific sequence AAPV. After elastase digestion two main products were observed for each peptide on RP-HPLC, and these were verified by MS. The first fragment corresponds to the active sequence and the enzyme recognition fragment. The second fragment is the same for all peptides (GGGQAGIVV) and covers the fibrillogenic part of peptides with a glycine linker. The amino acid sequences for all characterized fragments of the peptides are listed in Table 2.

In the next experiment all the studied peptides were incubated in PBS pH 7.4 for seven days to ensure fibril formation. After this time, the samples were mixed with neutrophil elastase and incubated together for 60 min. Finally, the obtained samples were analyzed by RP-HPLC and MS. As in the previous experiment, a digestion pattern was observed. On the RP-HPLC chromatograms (Figure 6B) a decrease in the substrate intensity was observed but, compared with non-fibrillated peptides, after 30 min of the experiment under the same conditions, the amounts of products were much smaller. This experiment showed that fibril formation significantly increased the digestion time and the accessibility of the peptide’s active form.

#### 2.2.2. Compound’s Stability in Plasma and Water

**Figure 7:**
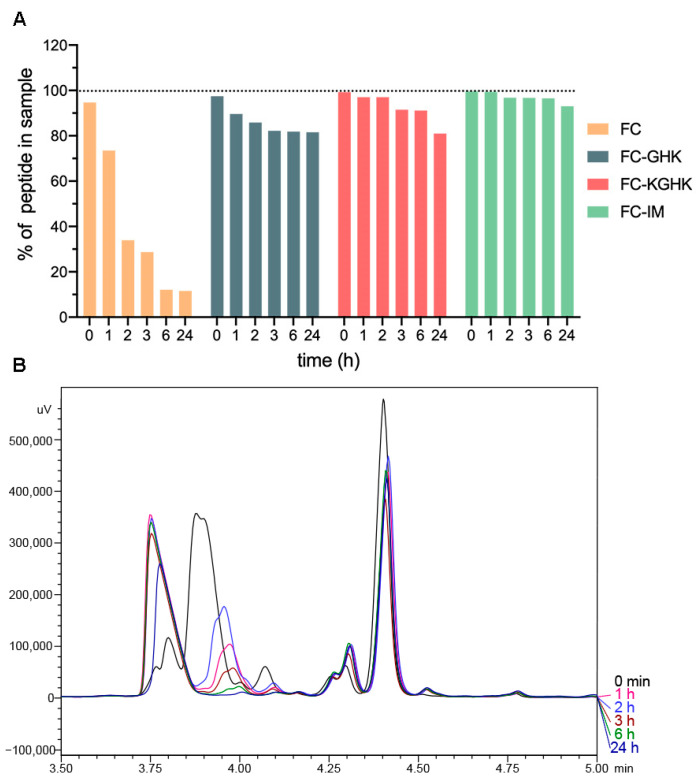
(**A**) Peptide stability with trichloroacetic acid (TCA) in human plasma and (**B**) fragment of the chromatogram (tR from 3.5 min to 5.0 min) of the FC peptide samples taken over 24 h of incubation.

In order to check the stability of the studied peptides in water and in human plasma, 24 h incubation of peptides in the appropriate medium was performed. The stability tests were based on the procedure described by Nguyen et al. [38]. In water, all the studied peptides displayed high stability (from 97 to 100% of peptide recovery after the incubation time (Figure S4)). Incubation in plasma led to moderate and case-dependent degradation of the studied compound (7% peptide loss for FC-IM, 20% peptide loss for FC-KGHK and FC-GHK, relative to the initial concentration, Figure 7A). The most significant changes were observed for FC, where only 11% of the peptide remained in the solution after 24 h incubation. The reason for this could be the visible and rapid precipitation of the FC peptide. In addition, chromatographic analysis of FC samples incubated in plasma (Figure 7B) revealed that decreased intensity of the signal of the FC peptide was accompanied by the appearance of a new signal with an earlier retention time. According to the mass spectrum analysis, the mass of a new compound (768 Da) was higher than that of the parent FC (584 Da). We suppose that the digested peptide fragments formed complexes but, due to the low importance of this process for our research, we did not pursue this observation further. The same phenomenon was not observed for FC incubated in water.

### 2.3. In Vitro and In Vivo Studies

#### 2.3.1. Cytotoxic Effects of Single Peptides

**Figure 8:**
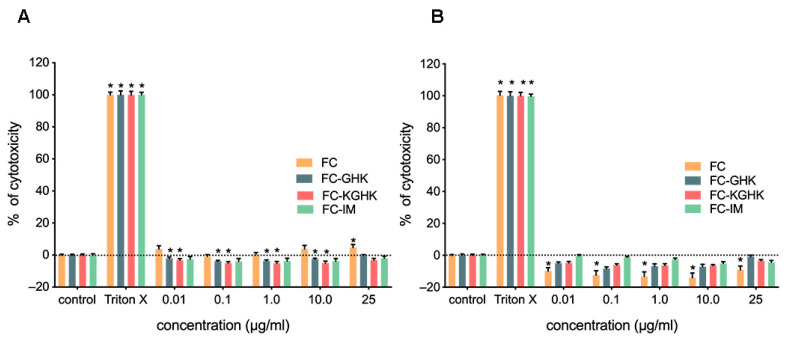
Scaffolds cytotoxicity toward, (**A**) 46BR.1N fibroblast and (**B**) HaCaT keratinocytes. The graphs show results from four independent experiments (four replicates in each, *n* = 16) for both tested cell lines. Results are presented as mean with SEM. *- statistically significant differences compared to the control, Mann-Whitney U test, *p* < 0.05. TRITON X-positive control cells incubated with medium containing 1% TRTION X, maximum LDH release = maximum cytotoxicity.

A cytotoxicity assessment of all the designed peptides was performed to evaluate their potential use in in vivo studies. The results of an LDH assay for all the tested peptides on immortalized human dermal 46BR.1N fibroblasts and HaCaT keratinocytes are shown in Figure 8A,B. Cytotoxicity studies showed that all the designed peptides were safe and did not cause a cytotoxic effect on fibroblasts or keratinocytes. Weak cytotoxicity (about 5%) against 46BR.1N fibroblasts was observed for FC at 25 µg/mL concentration.

#### 2.3.2. Human Skin Cell Proliferation by Single Peptides

**Figure 9:**
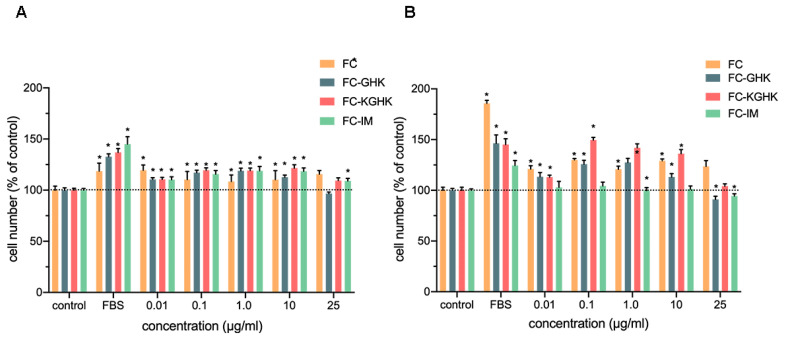
Peptide effect on proliferation of 46BR.1N fibroblasts after (**A**) 48 h and (**B**) 72 h incubation. The graphs show results from four independent experiments (four replicates in each, *n* = 16). Results are presented as mean with SEM. *- statistically significant differences compared to control, Mann-Whitney U test, *p* < 0.05. FBS-positive control cells grown in a medium containing 10% FBS.

**Figure 10:**
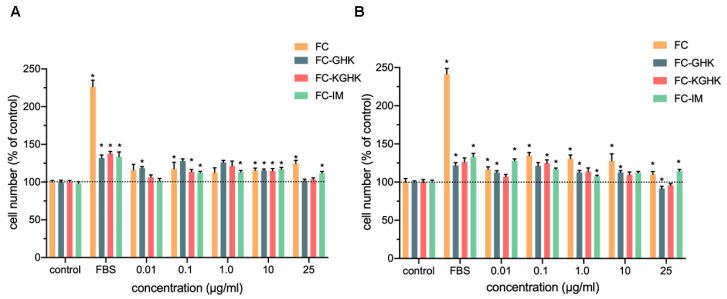
Peptide effect on proliferation of HaCaT keratinocytes after (**A**) 48 h and (**B**) 72 h incubation. The graphs show results from four independent experiments (four replicates in each, *n* = 16). Results are presented as mean with SEM. *- statistically significant differences compared to control, Mann-Whitney U test, *p* < 0.05. FBS-positive control cells grown in medium containing 10% FBS.

The XTT was used to evaluate the effect of the tested compounds on the proliferation of human 46BR.1N fibroblasts (Figure 9) and HaCaT keratinocytes (Figure 10). Analyses were performed after 48 and 72 h of incubation. The obtained results showed that the FC peptide stimulated proliferation of both tested cell lines (15–30% increase in proliferation compared to the control), and the effect was more substantial after 72 h incubation. Inhibition of the proliferation of both cell lines was not observed. The FC-IM peptide also caused an increase in proliferation of skin cells (20–30% compared to the control) but for the 46BR.1N fibroblasts, the effect was observed only after 48 h of incubation. Slight inhibition of the proliferation of fibroblasts was seen at a concentration of 25 µg/mL after 72 h incubation. FC-IM did not decrease the proliferation of HaCaT keratinocytes. The FC-GHK peptide stimulated proliferation of both tested cell lines at concentrations of 0.01–10 µg/L. For the 46BR.1N cells the effect was more robust after 72 h of incubation, while for HaCaT keratinocytes better activity was observed after 48 h. At the highest tested concentration, FC-GHK caused slight inhibition of the proliferation of skin cells after 72 h stimulation. FC-KGHK peptide showed pro-proliferative properties in both tested cell lines. The strongest stimulation was observed for 46BR.1N fibroblasts in concentrations of 0.1–10 µg/mL after 72 h of incubation. The effect was similar to that caused by FBS, which constitutes a positive control. For HaCaT keratinocytes the most potent stimulation was observed at concentrations of 1.0 µg/mL after 48 h and 0.1 µg/mL after 72 h incubation. However, the effect was slightly weaker than that obtained for fibroblasts. FC-KGHK did not inhibit the proliferation of skin cells.

#### 2.3.3. Collagen Synthesis Stimulation by Single Peptides

**Figure 11:**
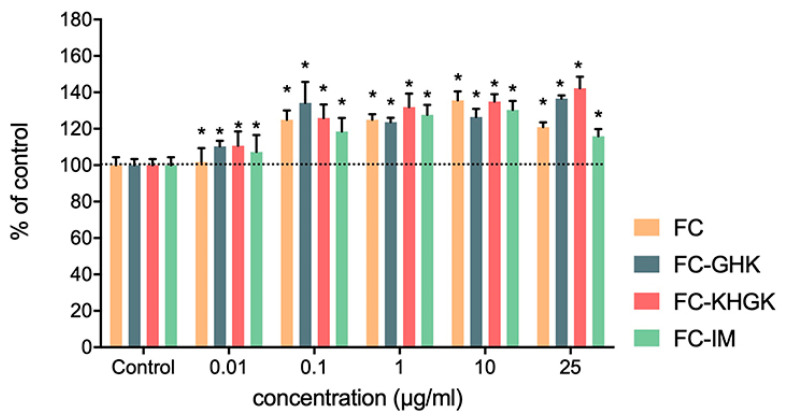
Effect of tested peptides on collagen synthesis in 46BR.1N fibroblasts. The graph shows results from three independent experiments. Results are presented as mean with SEM. *- statistically significant differences compared to control, Mann-Whitney U test, *p* < 0.05.

46BR.1N fibroblasts were stained with direct red 80 to analyze the effect of the tested peptides on collagen synthesis. The results showed an increase in collagen synthesis after stimulation with all the tested peptides (Figure 11 and Figure S5). A weak effect for all peptides (about a 10% increase in collagen synthesis) was observed at the lowest tested concentration. At concentrations of 0.1, 1.0 and 10.0 µg/mL, all the peptides caused a similar increase (20–30%) in collagen synthesis, while at 25.0 µg/mL FC-GHK and FC-KGHK showed the most substantial effect (30% and 40% increase, respectively). The observed effect is significant from a biological and clinical point of view. Proper synthesis and collagen production during the skin healing are essential for suitable skin strength and homeostasis in the skin.

#### 2.3.4. Cytotoxicity and Pro-Proliferative Effect of Peptide Fibril on Skin Cells

**Figure 12:**
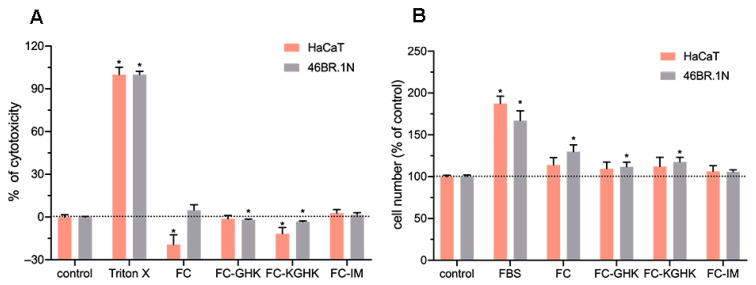
Effect of fibrils on human skin cells after 48 h of incubation. (**A**) Cytotoxicity of the scaffolds toward HaCaT keratinocytes and 46BR.1N fibroblasts; (**B**) Scaffold effect on proliferation of HaCaT keratinocytes and 46BR.1N fibroblasts. The graphs show results from three independent experiments. Results are presented as mean with SEM. *- statistically significant differences compared to control, Mann-Whitney U test, *p* < 0.05.

The LDH cytotoxicity analysis and the XTT proliferation test were performed for fibrilized scaffolds after 48 h of incubation. The conducted analyses showed that fibrils are not cytotoxic to human cells (Figure 12A). In addition, a statistically significant pro-proliferative effect was observed for FC, FC-GHK and FC-KGHK on 46BR.1N fibroblasts (Figure 12B). The most potent effect was visible for FC fibrils (30% increase, compared to control). FC-GHK and FC-KGH increased proliferation of 46BR.1N fibroblasts by about 10% and 20%, respectively. Statistically significant differences in the proliferation of HaCaT keratinocytes after stimulation with soluble FC, FC-GHK and FC-KGHK were not observed. Only a small (about 10%) increase in proliferation was noted for these fibrils. The FC-IM scaffold did not stimulate proliferation of either tested cell line.

### 2.4. In Vivo Studies

#### 2.4.1. Wound Healing in a Dorsal Skin Excision Model in Mice

**Figure 13:**
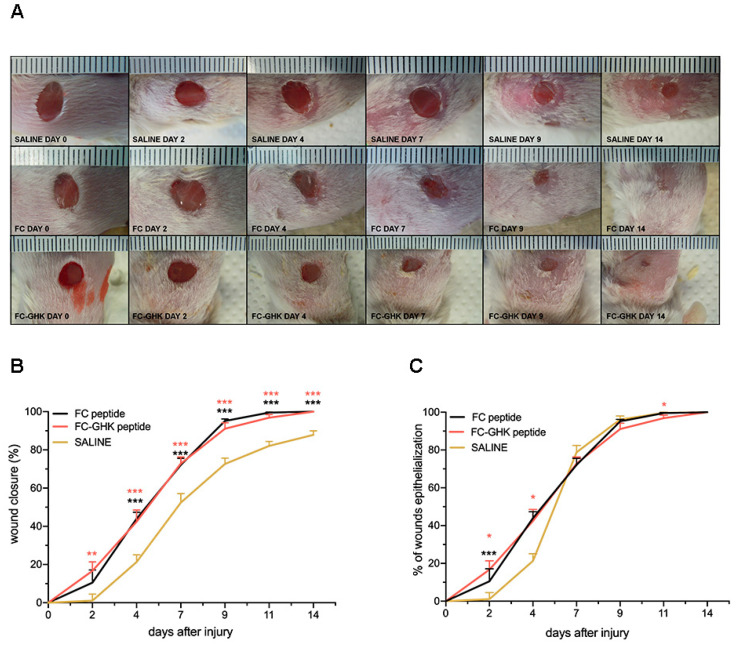
(**A**) Representative images of dorsal skin wounds in the control group and the non-fibrillated FC peptide and non-fibrillated FC-GHK peptide treatment groups. The images show wound healing progress from day 0 to day 14. (**B**) The relative area of dorsal skin injuries; (**C**) and wound epithelialization of the non-fibrillated FC and FC-GHK peptides compared to saline over time. The graph shows the average wound area relative to the initial wound surface, and error bars represent SEM. The number of wounds *n* = 12, representing six animals from both treatment groups and the control group. Statistical significance was determined with the Mann-Whitney U test; one (*), two (**), or three asterisks (***) indicate *p* < 0.05, *p* < 0.01, or *p* < 0.001, respectively.

We decided to conduct further studies in an in vivo mouse dorsal skin injury model, testing two selected peptides in non-fibrillated form and fibrillated form. The wound healing model of dorsal skin excision is a tissue repair model that has been found useful in testing the pro-regenerative activity of pharmacological agents [39]. The first was the FC peptide containing only a fibrillogenic sequence, intended to serve as a control. The second peptide was FC-GHK, as it forms peptide fibrils the fastest, did not cause any synthetic problems, and its biological activity in cellular tests is very similar to the other peptides tested. Additionally, FC-GHK stimulates fibroblasts to produce collagen to an appreciable extent. We first performed an in vivo experiment for the non-fibrillated FC and FC-GHK peptides. Both of the peptides were administered to the wounded mouse skin five times (at a 1 mg/mL concentration, in saline) every day during the first week. In total, each mouse received five applications of peptides to its wounds. The control group received only saline. The experiment was conducted over the course of 18 days, but due to the overgrown skin of the mice and no change in the overgrowth of the wound, plots were made until day 14. Representative images are presented in Figure 13A. On day 14, post-injury wounds were closed entirely in both treated groups, unlike the control group. Stimulation with the FC-GHK peptide resulted in faster wound closure than for the FC peptide and significantly smaller scars compared to the control group. Furthermore, in all groups, most healed skin was covered in fur by day 14, and the area was completely covered in fur by day 18. Changes in the relative wound area are presented in Figure 13B. The wound closure in both groups stimulated with FC and FC-GHK was significantly and consistently accelerated from day 2 till day 14, compared to the control group. Interestingly, in the early days of the experiment, we observed immediate wound closure in the groups stimulated with the FC and FC-GHK peptides, in contrast to the control group. We also observed similar wound epithelialization results in the groups treated with the FC and the FC-GHK peptides.

Epithelialization (Figure 13C) was more pronounced during the early stages, compared to the control group. Starting from day 7, the wound epithelialization process was identical for both the peptide-treated and control groups. This early epithelialization could be the result of systematic administrations of fresh portions of the peptides to the wound site. We obtained almost identical results for both peptides differing in their amino acid sequence, suggesting that the fibril peptide is sufficient for the wound healing effect. Although intended as a control group, the FC peptide without the active GHK sequence showed a marked healing activity.

**Figure 14:**
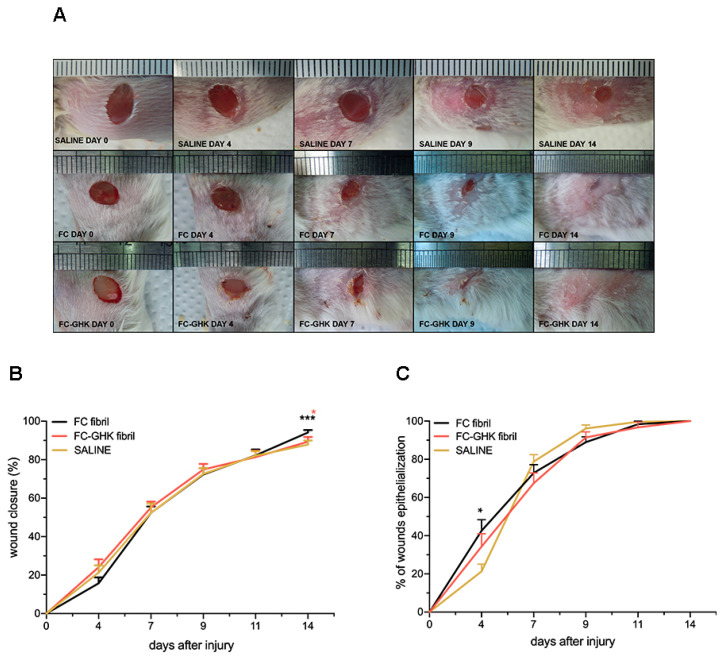
(**A**) Representative images of dorsal skin wounds in the control group and the groups treated with the fibrillated peptides FC and FC-GHK. The images show wound healing progress from day 0 to day 14. (**B**) The relative area of dorsal skin injuries and (**C**) and wound epithelialization of the fibrillated peptides FC and FC-GHK compared to saline over time. The graph shows the average wound area relative to the initial wound surface, and error bars represent SEM. The number of wounds *n* = 16 representing eight animals from both treatment groups and *n* = 12, representing six animals from the control group. Statistical significance was determined with the Mann–Whitney U test; one (*) and three asterisks (***) indicate *p* < 0.05 or *p* < 0.001, respectively.

Subsequently, we applied the peptides as fibrils. The fibrils were administered once at the start of the experiment (concentration was 1 mg/mL). The control group received only saline. The experiment was conducted over the course of 14 days, and representative images of healing skin are presented in Figure 14A. Similar to the previous experiment, wounds were closed entirely in both treated groups on day 14 post-injury. The photos show that wound healing was fastest in the group of animals treated with FC-GHK peptide. The wound closure and epithelialization process are shown in Figure 13B,C, respectively.

Contrary to the previous experiments, where wounds were treated with the non-fibrillated FC and FC-GHK peptides, the difference in wound closure rate could be observed on day 4 post-injury without statistical significance. Starting from day 4, the wound closure rate converged for all three groups, with the relative wound area becoming almost indistinguishable on day 7 post-injury. This phenomenon may be accountable to the fact that a single dose of fibrils might have been insufficient to produce the same results as free peptides administered in five doses.

The conducted experiments on the non-fibrillated peptides showed a potent healing effect on skin wounds from the first days of application, while fibrils displayed much weaker activity, if any. Rapid healing of the wound, mainly manifested by epithelialization, is critical in the first days of skin wound healing, especially in wounds with extensive damage area as it isolates the wound from environmental contaminants and pathogens [40].

#### 2.4.2. Tissue Isolation for Histological Analyses

**Figure 15:**
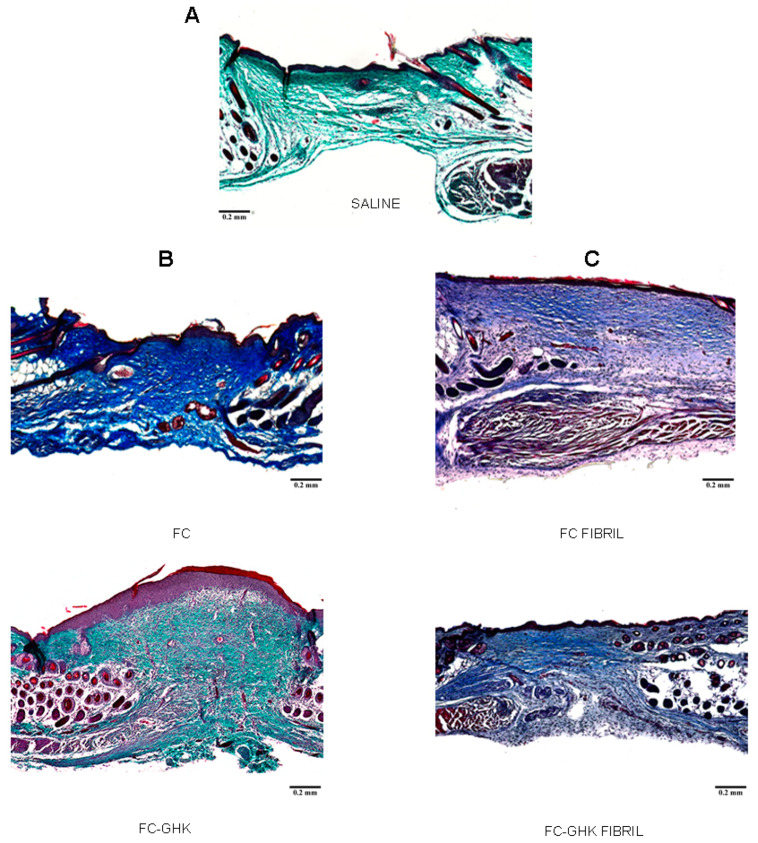
Representative images of histological samples on day 18 after injury: (**A**) saline; (**B**)-left panel non-fibrillated peptides FC and FC-GHK; (**C**)-right panel fibrillated peptides FC and FC-GHK. Skin samples stained with Masson’s trichrome. Scale bar 0.2 mm.

In order to investigate whether the FC and FC-GHK free peptides and fibrils affected the architecture of the restored tissues, we harvested skin samples on day 18 after injury. Figure 15 presents representative images of histological sections of murine dorsal skin taken from the wound site. All samples were stained using Masson’s trichrome method: the non-fibrillated peptides were stained with light green, and the fibrillated ones with aniline blue. In the control group—treated only with saline—the newly formed tissue was condensed under the epidermis, with deeper layers being loose and unorganized. For the skin samples stimulated with the FC-GHK peptide (Figure 15B), a thicker layer of more uniformly distributed cells can be seen. More instances of newly formed hair follicles, a high density of fibroblasts, and a more uniform cell distribution were observed in comparison with the control group. Tissue stimulated with the FC-GHK showed a modest reconstruction of the muscle cell layer, a phenomenon absent in both the FC-stimulated and the control tissues.

The histological sections from the wounds treated with peptide fibrils (Figure 15C) show a central scar with little to no appendage formation. However, in the FC-treated group, a substantial outgrowth of muscle cells can be noted. These muscle cells grew into the wound area in a mostly disorganized manner and did not appear to form a layer. In one case, these cells formed an organized layer that stretched across the entire wound area. No such cell growth was present in FC-GHK treated skin samples, thus suggesting that the FC fibril may stimulate smooth muscle cell growth or cell movement after an injury.

## 3. Discussion

Functional scaffolds for application in regenerative medicine should possess several desirable features such as: biophysical and mechanical properties that mimic the microenvironment of healthy tissue (typically the extracellular matrix); an ability to form a 3D porous network that promotes cell growth by allowing the transport of nutrients and metabolic waste in and out of the scaffold; and tunable biodegradability, allowing the rate of scaffold degradation to match that of new tissue growth [41,42,43,44]. All the features mentioned above are possessed by the peptide fibrils that are the subject of this publication. We tested peptide fibrils for application in regenerative medicine, specifically for skin wound healing. We added biologically active sequences to peptide fibrils, to check if they could accelerate wound healing. Skin wounds are one of the problems associated with civilization diseases and one of the problems of people after accidents and extensive burns. The deficit of effective pharmacological solutions creates a need to search for new skin wound healing agents.

An example of a peptide fibril used in regenerative medicine applications is the IKVAV peptide [1]. This sequence corresponds to the neurite-promoting laminin epitope, which, when constituted into nanofibrils, has been shown to induce very rapid differentiation of cells into neurons, at a better rate than soluble peptide or laminin itself [45]. Li et al. combined the sequence of IKVAV with the sequence RGD to obtain a self-assembly compound that was shown to induce better cell proliferation [46]. This peptide also supports and guides axonal regeneration without morbidity and functional loss. In addition, a nanofiber hydrogel’s ability to regenerate collagen in response to cavernous nerve injury in prostatectomy and diabetic patients has also been reported, providing another promising use for amphiphilic peptides that form hydrogels in regenerating tissues [47].

Our research focused on a short fibrillogenic peptide that can be used as a scaffold for pro-regenerative active sequences. For this purpose we chose a peptide with a proven aggregation properties and corresponds to the fragment of the steric zipper sequence from a human protein—cystatin C—to ensure the effective fibrillization of the final hybrid compound [31,32]. The aggregation tests, ThT assay and microscopic techniques (TEM and AFM) confirmed that the FC peptide forms stable fibrils, and thus can be a scaffold for sequence modifications and further research. We then included this fibrillogenic fragment in new hybrid compounds, which also contained an elastase-specific sequence (AAPV) and three pro-regenerative sequences: (i) GHK [36,37]; (ii) KGHK [34] and (iii) RDKVYR [33]. Aggregation tests were also performed for these peptides. The obtained results showed that all peptides, similar to the FC peptide, undergo fibrillation immediately after dissolution in phosphate buffer, but the FC-GHK peptide forms fibrils most efficiently. Elongation of the core of the fibrillogenic sequence by additional, hydrophilic residues, changes the fibrillization rate and, slightly, the morphology of the fibrils. At the same time, this does not abolish the scaffold formation, which is very important from the point of view of further studies.

Release of the active sequence from the scaffold occurs on enzymatic digestion with elastase. We chose this enzyme because of its prominent role in the degradation of proteins and reduction in the quantity of bacteria at the damaged tissue’s location [34,48]. A high concentration of this enzyme has been found in body fluids of inflammation wounds [49,50]. In all peptides, we included a specific sequence for elastase. A similar approach has successfully been used in studies of the release of active sequences, such as RGD or growth factor TGF-β, from hydrogels like RADA16, using metalloproteinase 2 [51,52,53,54,55]. The enzymatic digestion experiments performed for the FC-GHK, FC-KGHK and FC-IM peptides showed that the enzyme cleaved all peptides into two fragments. The rate of enzymatic cleavage depends on whether the peptide is in a non-fibrillated or fibrillated form. We expected that the digestion process of the fibrils would be much longer than for the free peptides, but we observed that the difference was not striking (a twofold increase). This is most likely because, in the studied peptides, FC-GHK, FC-KGHK and FC-IM, the elastase-sensitive and active sequences are not included in the fibrillar core but are to some extent available to the enzyme. Glycine is an amino acid that typically introduces great conformational freedom and increases the conformational entropy of peptides [56]. Thanks to this, the enzyme probably has free access to the sensitive sequence. However, in our work, we proved that the peptide fibril was more resistant to the enzyme than the peptide itself.

Our studies confirmed that the analyzed hybrid peptides were stable in water and plasma. In comparison to the unmodified FC peptide, which is rapidly degraded in plasma, attachment of additional amino acid residues increased the stability of the peptides enough to make these peptides reasonable candidates for subsequent cytotoxicity studies and the assessment of their pro-regenerative potential.

We conducted biological tests to confirm the safety of the designed compounds (cytotoxicity analysis) and evaluate their potential to stimulate of wound healing. In these experiments we examined the non-fibrillated and fibrillated peptides separately. As an in vitro model, we chose immortalized human cell lines, namely HaCaT keratinocytes and 46BR.1N fibroblasts, which constitute a reliable model for the initial assessment of peptide activity in wound healing. This model was successfully used to evaluate the activity of different peptides, e.g., IM or PDGF peptide derivatives, in our previous works [33,57,58]. The analyses performed using the LDH test did not show any cytotoxic effect for any of the tested free peptides or fibrils. Low cytotoxicity was expected for the studied peptide fibrils, because similar results were obtained for amyloid fibrils derived from short peptides [59]. For example, lysozyme-derived amyloid fibrils were shown to be non-toxic to L929 fibroblasts and increased their proliferation [60]. Similarly, amyloid-based hydrogels composed of amyloid nanofibrils designed from the C-terminus of amyloid-β (Aβ) protein were proved to be non-toxic to cells and support growth and spreading of L929 fibroblasts and human mesenchymal stem cells (MSCs) [61]. When analyzing cultured skin cells, we noticed that the FC peptide, has a pro-proliferative effect, especially in its fibrillar form. These results showed that each peptide can be biologically active, although it was not designed for this application.

Collagen is a crucial protein taking part in wound healing of skin and other tissues, and its synthesis is necessary to restore the mechanical strength of skin [62]. We evaluated the effect of designed peptides on collagen synthesis by 46BR1.N fibroblasts and found that all the tested peptides stimulate collagen synthesis to a different extent. The results of in vitro analyses of the designed peptides confirmed their safety and the fact that these compounds can be used to promote skin wound healing.

In vivo tests on Balb/C mice in an excisional skin wound model were performed for the FC and FC-GHK peptides, in their non-fibrillated and fibrillated forms. Both of these peptides significantly accelerated skin wound closure compared to controls administered saline alone. A better effect of FC-GHK, in comparison to FC, was recorded on the second day of wound closure. In addition, both peptides showed increased epithelialization on the second day, in comparison with the control. This process is critical because the first days after the injury are crucial in the healing process preventing bacterial infection [63]. The positive effect of wound healing after administering the FC-GHK peptide was also visible in histological sections collected 18 days after the injury. These observations suggested that the addition of the GHK sequence to the scaffold peptide resulted in a slightly better regenerative effect. In conclusion, both peptides significantly accelerated the wound closure process and are good candidates as pro-regenerative compounds. It is worth noting that the speed of skin wound closure or epithelialization and the quality of the reconstructed tissue presented in this work are similar to the effects reported by other researchers for peptide hydrogels [64], short peptides from frog skin [65] and other peptides [66,67].

The addition of an active sequence (GHK) to FC did not affect the wound closure rate. The fibrillated peptides insignificantly accelerated the wound epithelialization process on the first day after the injury, compared to the control, but the fibrils were not as effective at epithelialization as the free peptides. In contrast to the soluble FC and FC-GHK peptides, the fibrillated forms showed weaker wound healing effects, yet the first were administered in five doses, while the latter were given in a single delivery. Nevertheless, the reduced activity of the fibrils may result from the release of active peptides in the wound environment being too slow, and perhaps the fibrils may be found more effective in treating chronic wounds. We also observed that the application of FC fibrils led to formation of a significant layer of muscle cells under the collagen deposition. This effect warrants further examination in the context of muscle regeneration (gene activations, immunohistochemistry, transcriptomics, etc.). This is, however, beyond the scope of the current study. We found no reports in the literature on the study of non-hydrogel-forming peptide fibrils as agents for promoting wound healing. To our knowledge, this is the first experiment of this type yet described.

## 4. Materials and Methods

### 4.1. Peptide Synthesis, Purification and Ion Exchange

Peptides were synthesized by the standard solid phase method using N-Fmoc protected amino acids, using a Liberty Blue^®^ automated microwave synthesizer (CEM Corporation, Matthews, NC, USA). Fmoc Rink Amide (LL) SpheriTide (CEM Corporation, Matthews, NC, USA) resin was used as a solid support. For the synthesis by Fmoc methodology, standard reagents were used, such as 20% piperidine solution in DMF, 0.5 M DIC solution in DMF, 1.0 M solution of Oxyma Pure^®^ and 0.1 M DIPEA in DMF. Peptides were purified by preparative high-performance liquid chromatography on a reversed phased (RP-HPLC) column (Jupiter^®^ Proteo C12, 4 μm, 90 Å, 21.2 × 250 mm, Phenomenex, Torrance, CA, USA) and analyzed using analytical RP-HPLC (Jupiter^®^ C18 column, 5 μm, 300 Å, 4.6 × 250 mm, Phenomenex, Torrance, CA, USA), and mass spectrometry (ESI LCMS IT TOF, Shimadzu, Kyoto, Japan). A linear gradient of 80% acetonitrile (B) was applied as a mobile phase. The purified peptides, obtained as trifluoroacetate salts, were subjected to counter-ion exchange to the acetate form.

### 4.2. Fibril Preparation

All peptides were dissolved in Eppendorf Low Retention Tubes in 1.0 mL of PBS pH 7.4. The concentration of the solutions of peptides was 1.0 mg/mL. The peptides were incubated at 37 °C with constant orbital shaking. Aliquots for the evaluation of the fibrillization process were taken before incubation and after 1, 2, 3, 6, 14, and 21 days or only after 7 days, depending on the experiments.

### 4.3. Thioflavin T Fluorescence Assay

Ten microliters of peptide solution incubated under fibrillization conditions (see Fibril preparation) were mixed with 10 µL of 1.5 mM ThT in water and 90 µL of PBS pH 7.4. The fluorescence was measured on a Tecan Infinite 200Pro (Tecan Group Ltd., Männedorf, Switzerland) spectrofluorometer in 96-well plates (Costar Black^®^), with excitation wavelength set at 420 nm and emission in the range of 455 to 600 nm.

### 4.4. Atomic Force Microscopy

Analyzed samples (peptide fibrils), with a concentration of 0.001%, were prepared in Milli-Q water. A small drop of the tested peptide was applied to a freshly cleaned mica surface, rinsed after 30 s with 100 μL of water and left to dry in air. The topography of deposited samples was analyzed using a JPK NanoWizard^®^ 4 atomic force microscope (Bruker Nano GmbH, Berlin, Germany). The measurements were performed in QI (Quantitative Imaging Mode) using Tap150AL AFM cantilevers (Ted Pella, Inc., Redding, CA, USA). The AFM images were processed and analyzed using the Gwiddion 2.49 program package [68].

### 4.5. Transmission Electron Microscopy

Peptide samples (5 µL) were applied on a glow-discharged carbon-coated copper grid (400 mesh). After 1 min of adsorption, excess liquid was removed using filter paper, and the samples were stained with 2% (*v*/*v*) aqueous uranyl acetate. The samples were examined with a TECNAI SPIRIT BIO TWIN FEI (FEI Company, Hillsboro, OR, USA) at 120 kV, with nominal magnifications between 11,500 and 39,000. The assay was performed for samples before (after dissolution) and after 3 days of incubation.

### 4.6. Circular Dichroism

Circular dichroism spectra were recorded on a Jasco J-815 spectropolarimeter (Jasco, Victoria, BC, Canada). Far-UV spectra (195–250 nm) were registered for all peptides dissolved in PBS pH 7.4 at a concentration of 1.0 mg/mL and incubated at 37 °C with agitation. Spectra were recorded before and after 1,2, 3, 7, 10 and 14 days of incubation. All spectra were corrected for the buffer signal. The CD spectra were recorded three times, using a 0.2 mm cell.

### 4.7. Incubation with Neutrophil Elastase for Peptides and Fibrils

An enzymatic stability study was performed using the human neutrophil elastase enzyme (Sigma Aldrich, St. Louis, MO, USA). The peptides were dissolved in a buffer (with composition: 0.1 M Tris HCl, 500 mM NaCl, 0.05% Triton X-100, 20 mM CaCl_2_, pH 7.5), to a final concentration 1.0 mM. The fibrils formed in the aggregation process were dissolved in PBS at pH 7.4 to a concentration of 1 mg/mL. The enzyme was added to the peptide/fibril solution to a concentration of 150 nM and incubated for 30 min (peptides) or 60 min (fibrils), at 37 °C, with agitation. A 10% aqueous TFA solution was added to the reaction mixture to stop the digestion. The progress of the reaction was monitored by RP-UHPLC with a PDA (Photo Diode Array) detector on a Kinetex C8 column (2.6 μm, 100 Å 2.6 × 100 mm, Phenomenex, Torrance, CA, USA), at a gradient of 0–50% B for 15 min, and by LC-MS.

### 4.8. Peptides and Fibrils Stability in Water and Human Plasma

Peptide stability tests were performed according to a TCA stability assay based on the method used by Nguyen et al. [38]. Peptides were dissolved in water, mixed with human plasma* to a final concentration of 1.0 mg/mL, and incubated at 37 °C with agitation (300 rpm, Thermomixer, Eppendorf AG, Hamburg, Germany). After 0, 1, 2, 3, 6 and 24 h, 80 μL of the solution was taken and mixed with 15% TCA to obtain a final concentration of 3% (*v*/*v*). The samples were incubated in ice for 10 min and centrifuged for 10 min (12,000 rpm, Microfuge 16, Beckman Coulter, Brea, CA, USA). Supernatants were analyzed by RP-HPLC on column using a linear gradient of acetonitrile (5–100% over 15 min) with detection at 223 nm. Peptides were quantified by their peak areas relative to the initial peak areas (0 min). All stability tests were performed at least in triplicates. As control samples peptides dissolved only in water under the same conditions were used. To additionally assess the changes in the incubated samples (peptide degradation) mass spectrometry analysis was used. LC-MS experiments were performed using ESI-IT-TOF (Shimadzu, Kyoto, Japan).

* Blood was collected from healthy anonymous donors and EDTA was then used as an anticoagulant. The procedure was approved by the Independent Bioethics Commission for Research of the Medical University of Gdańsk (NKBBN/387/2014).

### 4.9. Cell Culture Conditions

Immortalized human HaCaT [69,70] keratinocytes (DKFZ, Heidelberg, Germany) and the human dermal fibroblast cell line 46BR.1N (ECACC, Sigma Aldrich, St. Louis, MO, USA) were used in the study. Cells were routinely grown in culture flasks (growth surface area 25 cm^2^) in Dulbecco’s modified Eagle medium (DMEM, Sigma Aldrich, St. Louis, MO, USA), with 4500 mg/L of glucose, 584 mg/L of L-glutamine, sodium pyruvate, and sodium bicarbonate, supplemented with 10% FBS and 100 units/mL of penicillin and 100 μg/mL of streptomycin (Sigma Aldrich, St. Louis, MO, USA), under a humidified atmosphere with 5% CO_2_ at 37 °C. Medium was changed every 2–3 days.

### 4.10. LDH and XTT Assays

Lactate dehydrogenase (LDH) activity in cell culture supernatants (Takara, Japan, cat. No. MK401) was measured to determine the cytotoxicity of the tested compounds, and the XTT assay was used to evaluate their effect on cell proliferation. Both tests were performed as previously described [33,57] according to manufacturers’ instructions. Briefly, cells were seeded into 96-well plates, at a density of 5000 cells per well, in DMEM supplemented with 10% FBS. After 24 h the medium was exchanged for serum-free DMEM containing appropriate concentrations of the tested compounds. Supernatants for LDH analysis were collected after 48 h of incubation. The XTT assay was performed after 48 and 72 h of incubation.

For the analysis of the effect of the peptide fibrils on skin cells, the experiment was performed as described, but the cells were stimulated with 10 µL of the fibrils (50 µg/mL) and incubated for 48 h, when the LDH and XTT assay were then performed.

### 4.11. Collagen Synthesis Analysis

46BR.1N fibroblasts were seeded into 96-well plates, at a density of 3000 cells per well, in DMEM medium supplemented with 10% FBS. After 24 h, when the cells had attached to the plate, the medium was exchanged for a fresh one, and the cells were stimulated with appropriate concentrations of the tested peptides. Half of the medium was exchanged every two days, with additional stimulation during every medium exchange, and the cells were cultured in the presence of the peptides for six days. The medium was then removed and the cells were washed with PBS, fixed for 1 h in Bouin’s fluid (15 mL of saturated picric acid, 5 mL of 37% formaldehyde and 1 mL of acetic acid), washed in running tap water for 15 min and air dried. The cells were stained for 1 h with Direct red 80 (Sigma Aldrich, St. Louis, MO, USA) dissolved in saturated aqueous picric acid solution at a concentration of 1 mg/mL, while gently shaking the plate on a microplate shaker. The dye was removed and the cells were washed with a 0.01 M hydrochloric acid solution to remove unbound dye. The cells were photographed with a light microscope. The dye was then dissolved in 0.1 M sodium hydroxide for 30 min by shaking the plate on a microplate shaker at room temperature. The solution was transferred to a new plate and a spectrophotometric analysis was performed at 550 nm, using 0.1 N sodium hydroxide as a blank [71]. The results are presented as percentage of control.

### 4.12. Wound Healing Model in Mice

Eight-week-old BALB/c female mice were used for experiments. All experimental procedures were approved by the Local Ethics Committee in Bydgoszcz (Approval No. 49/2016). The mice were anesthetized with inhaled 2–5% isoflurane. The skin on the back was shaved and disinfected. The skin was folded and raised cranially and caudally along the spine. The mouse was then placed in a lateral position, and the folded skin was pierced with a 6.0 mm diameter biopsy punch, resulting in the formation of two excisional, symmetrical wounds on the back of the mouse. Before peptides administration, mice were randomly divided into treatment and control groups comprising six individuals. A total of 10 µL of 1 mg/mL peptide solution suspended in saline or fibrils solution suspended in PBS at the same concentration, was applied to each wound. Control groups received saline only. The wounds were covered with transparent Tegaderm film and an adhesive plaster was wrapped around the mouse torso. During the first week of the experiment, the wounds were treated with the peptide daily for five consecutive days along with replacement of the dressing. During the second week of the experiment, the dressing was replaced every other day with no further stimulation. For fibrils the solution was administered once after wounding and the dressing was replaced every two days. At the beginning of the third week, the dressing was removed. To measure the wound area, a ruler was placed next to the injury site, and the wound was photographed. Wound areas were calculated using ImageJ software [72].

### 4.13. Tissue Isolation for Histological Analyses

Mice were euthanized on day 18 after the injury. Skin from their backs was sampled and stored in formalin. Samples were then embedded in paraffin, cut into 5 µm sections and stained with Masson’s trichrome staining (light green for control and FC-GHK samples and aniline blue for the FC peptide and both fibrillated peptides). Stained sections were evaluated using a light microscope.

### 4.14. Statistical Analysis

Statistical significance was determined with the Mann–Whitney U test; one (*), two (**), or three asterisks (***) indicate *p* < 0.05, *p* < 0.01, or *p* < 0.001, respectively, using STATISTICA 13.3 software (StatSoft, Warsaw, Poland) [73]. Graphs were prepared with GraphPad Prism 8.0.0 software (GraphPad Software, San Diego, CA, USA) [74].

## 5. Conclusions

In summary, we proved that the QAGIVV (FC) peptide could be a very promising fibrillogenic scaffold for active substances. This peptide has only six amino acid residues but is useful in driving the fibrilization process. The connection of FC with pro-regenerative sequences like GHK, KGHK and RDKVYR does not reduce this feature and, at the same time, does not impair biological activity. The most promising as a scaffold turned out to be the FC-GHK peptide. This hybrid peptide displayed pronounced propensities toward fibrillization, low cytotoxicity and significant pro-proliferative effects on human skin cells, and it accelerated dorsal skin wound healing in an excisional skin wound model when applied as a free peptide. It is worth noting that the FC peptide without the GHK modification displayed similarly significant skin wound healing activity. Our research showed that peptide scaffolds might become a safe alternative to traditional drug treatment. However, further research is necessary to investigate the mechanism of degradation or free peptide release from fibrils in in vitro models. In addition, the mechanism of this skin wound healing effect remains to be examined.

## Figures and Tables

**Table 1:** Amino acid sequences of the designed peptides.

Peptide ID	AMINO ACID SEQUENCE
**FC**	NH_2_-QAGIVV-NH_2_
**FC-GHK**	Ac-GHK-GGG-AAPV-GGG-QAGIVV-NH_2_
**FC-KGHK**	Ac-K GHK-GGG-AAPV-GGG-QAGIVV-NH_2_
**FC-IM**	Ac-RDKVYR GGG-AAPV-GGG-QAGIVV-NH_2_

**Table 2:** The amino acid sequences and masses of the digested fragments for studied peptides after peptide degradation by human neutrophil elastase.

Peptide ID	Degradation Yes/No	Amino Acid Sequences of Digested Fragments	*m*/*z*
FC-GHK	Yes	Ac-GHKGGGAAPV-COOH	892.6
		NH_2_-GGGQAGIVV-NH_2_	755.9
FC-KGHK	Yes	Ac-KGHKGGGAAPV-COOH	1020.2
		Ac-GHKGGGAAPVG-COOH	1077.2
		NH_2_-GGGQAGIVV-NH_2_	755.9
FC-IM	Yes	Ac-RDKVYRGGGAAPV-COOH	1345.5
		Ac-RDKVYRGGGAAPVG-COOH	1402.6
		NH_2_-GGGQAGIVV-NH_2_	755.9

## Data Availability

The authors were unable to find a valid data repository for the data used in this study. These data are available from Sylwia Rodziewicz-Motowidło at the University of Gdańsk, Gdańsk Poland (s.rodziewicz-motowidlo@ug.edu.pl).

## Supplementary Materials

The following are available online at https://www.mdpi.com/article/10.3390/ijms22083818/s1, Figure S1: Examples (A) Chromatogram and (B) mass spectra for the FC-GHK peptide after purification process; Figure S2: Photos of the precipitated peptides (FC, FC-GHK, FC-KGHK, FC-IM) obtained after seven days incubation; Figure S3: Mass spectra for FC-GHK after 30 min of incubation with neutrophil elastase; Figure S4: Peptide stability in water over 24 h incubation; Figure S5: Representative images of Direct red 80 staining of collagen (red areas) in 46BR.1N stimulated with the tested peptides.
