# Supplementary material for: Functionalized Peptide Fibrils as a Scaffold for Active Substances in Wound Healing
Source: Int J Mol Sci. 2021 Apr 7;22(8):3818. doi: 10.3390/ijms22083818 (PMC8067766; doi:10.3390/ijms22083818)
Supplement: Supplementary file 1 [file ijms-22-03818-s001.pdf]

International Journal of
Molecular Sciences

MDPI

Supplementary materials

Functionalized Peptide Fibrils as a Scaffold for Active Substances in Wound Healing

Justyna Sawicka [1,†], Emilia Iłowska [2,†], Milena Deptuła [3], Paweł Sosnowski [4], Piotr Sass [4], Katarzyna Czerwiec [5], Klaudia Chmielewska [3], Aneta Szymańska [1], Zuzanna Pietralik-Molińska [6], Maciej Kozak [6], Paweł Sachadyn [4], Michał Pikuła [3,*] and Sylwia Rodziewicz-Motowidło [1,*]

[1] Department of Biomedical Chemistry, Faculty of Chemistry, University of Gdańsk, 80-308 Gdańsk, Poland; justyna.sawicka@ug.edu.pl (J.S.); aneta.szymanska@ug.edu.pl (A.S.)
[2] Department of Organic Chemistry, Faculty of Chemistry, University of Gdańsk, 80-308 Gdańsk, Poland; emilia.ilowska@ug.edu.pl
[3] Laboratory of Tissue Engineering and Regenerative Medicine, Department of Embryology, Medical University of Gdańsk, 80-210 Gdańsk, Poland; milenadeptula@gumed.edu.pl (M.D.); klaudia.chm@gmail.com (K.C.)
[4] Laboratory for Regenerative Biotechnology, Faculty of Chemistry, Gdańsk University of Technology, 80-233 Gdańsk, Poland; paw.sosno@gmail.com (P.S.; Paweł Sosnowski); piotrsass@gmail.com (P.S.; Piotr Sass); psach@pg.edu.pl (P.S.; Paweł Sachadyn)
[5] Department of Clinical Anatomy, Medical University of Gdańsk, 80-210 Gdańsk, Poland; katarzyna.czerwiec@gumed.edu.pl
[6] Department of Macromolecular Physics, Faculty of Physics, Adam Mickiewicz University in Poznań, 61-712 Poznań, Poland; zuzannap@amu.edu.pl (Z.P.-M.); mkozak@amu.edu.pl (M.K.)
* Correspondence: pikula@gumed.edu.pl (M.P.); s.rodziewicz-motowidlo@ug.edu.pl (S.R.-M.); Tel.: +48-58-3491368 (M.P.); +48-58-5235037 (S.R.-M.)
† Equal contribution.

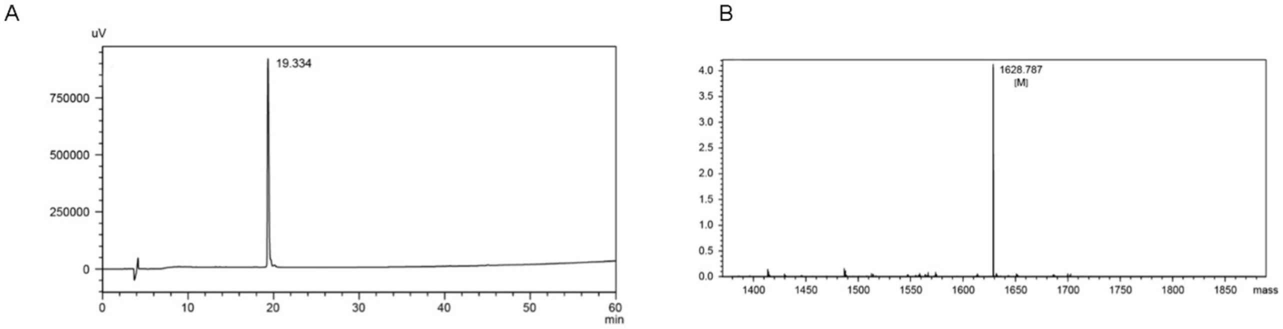

Figure S1. Examples of (**A**) Chromatogram and (**B**) mass spectra for the FC-GHK peptide after purification process.

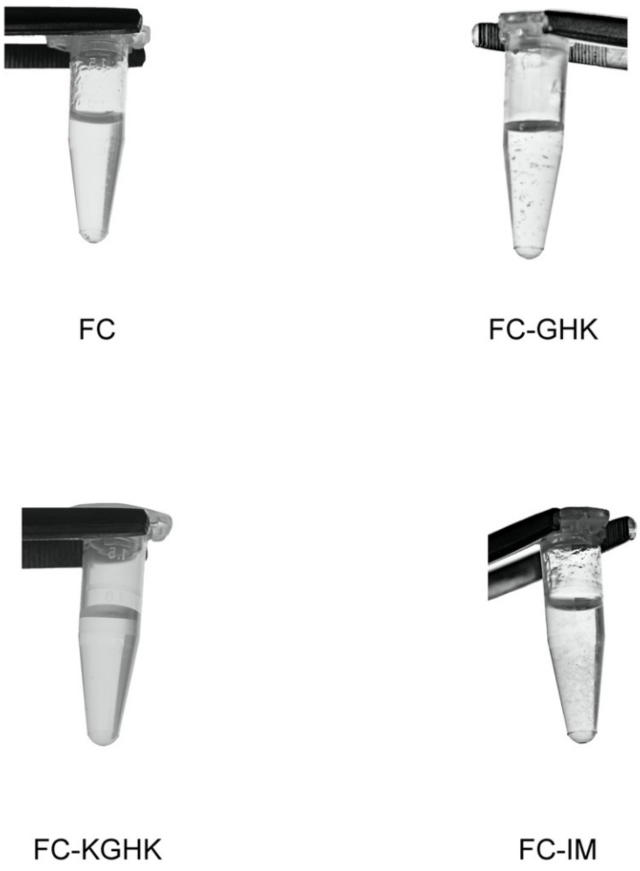

Figure S2. Photos of the precipitated peptides (FC, FC-GHK, FC-KGHK, FC-IM) obtained after seven days of incubation.

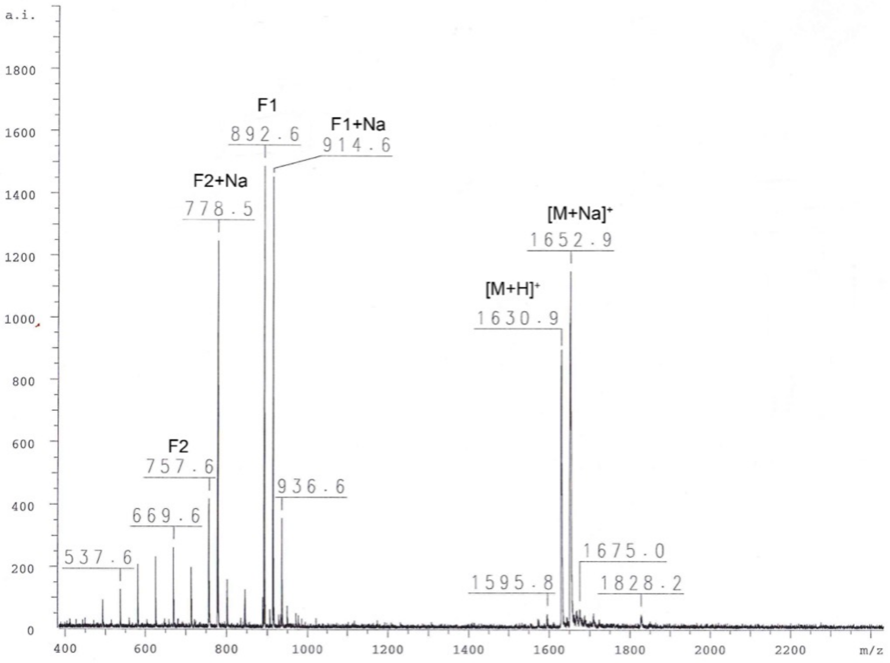

Figure S3. Mass spectra for FC-GHK after 30 min of incubation with neutrophil elastase.

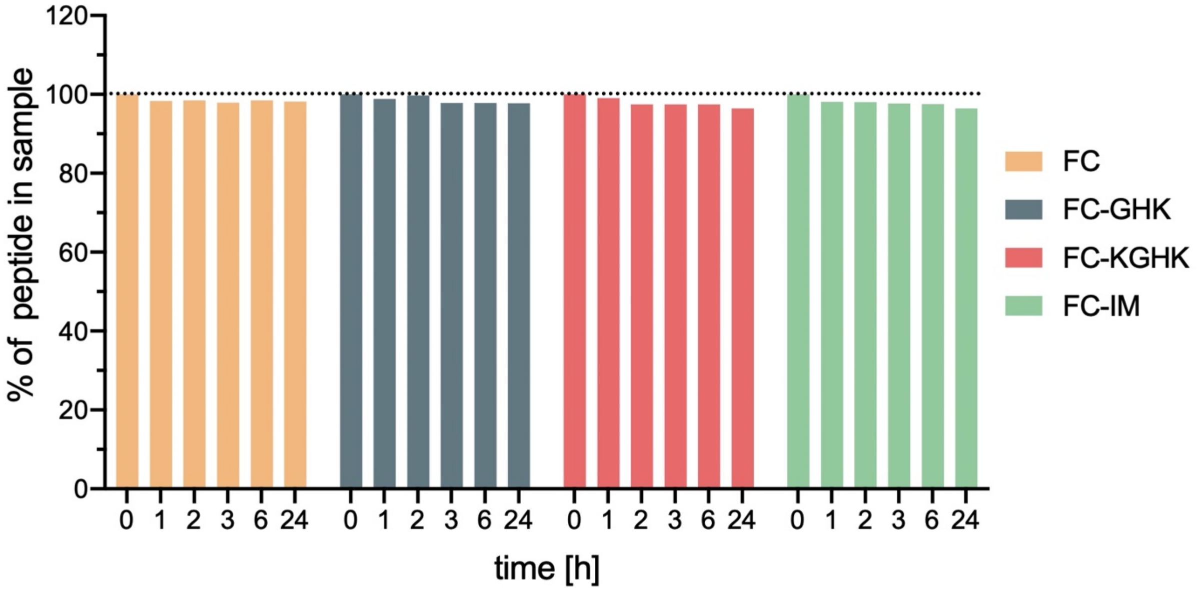

Figure S4. Peptide stability in water over 24 h incubation.

International Journal of
Molecular Sciences

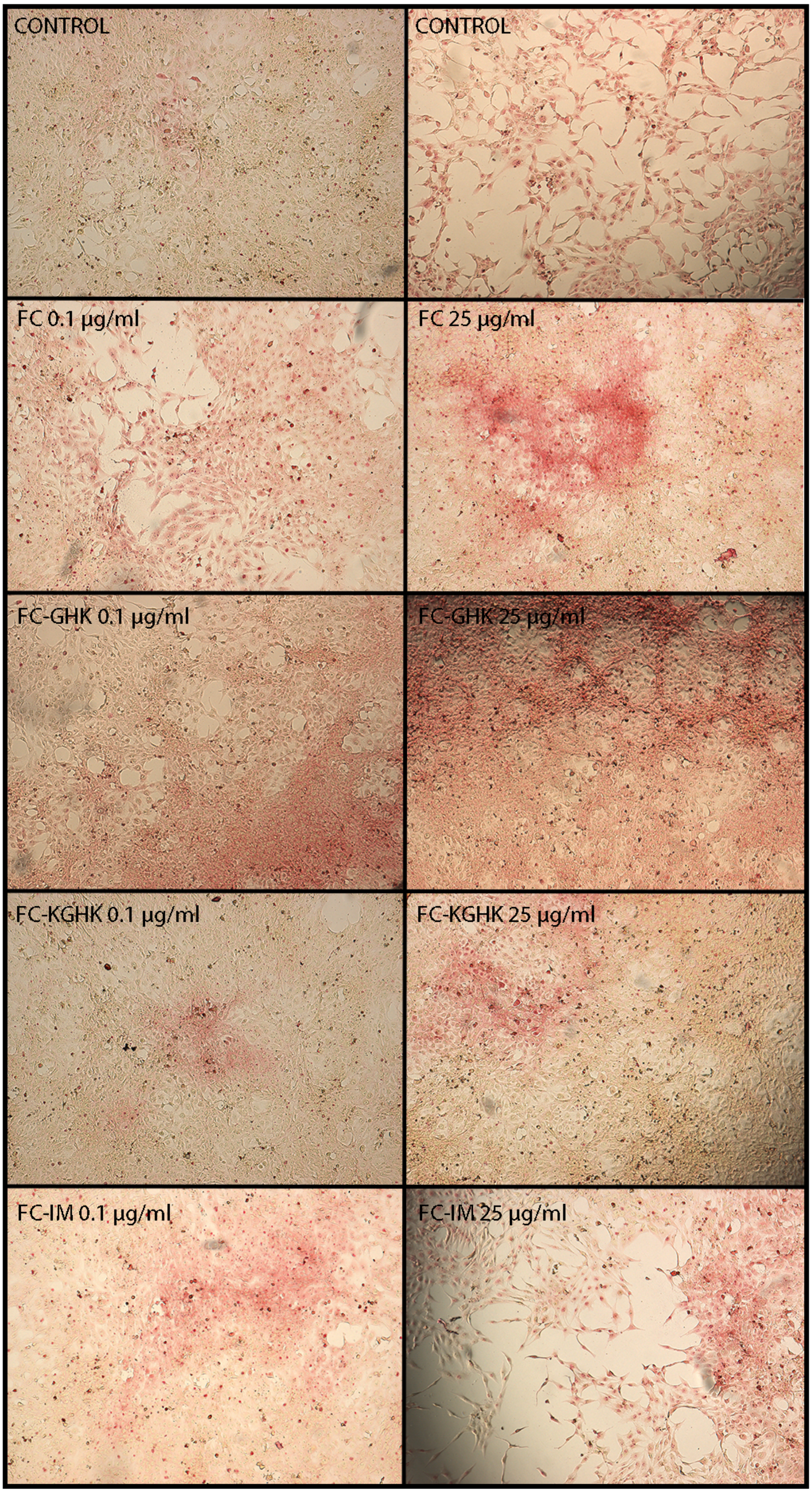

Figure S5. Representative images of direct red 80 staining of collagen (red areas) in 46BR.1N cells stimulated with the tested peptides.
